# *In Vitro* Acoustic Characterization of Three Phospholipid Ultrasound Contrast Agents from 12 to 43 MHz

**DOI:** 10.1016/j.ultrasmedbio.2013.10.010

**Published:** 2014-03

**Authors:** Chao Sun, Vassilis Sboros, Mairead B. Butler, Carmel M. Moran

**Affiliations:** ∗ Medical Physics, Centre for Cardiovascular Science, University of Edinburgh, Edinburgh, United Kingdom; † Institute of Biochemistry, Biophysics and Bioengineering, Heriot-Watt University, Edinburgh, United Kingdom

**Keywords:** High-frequency ultrasound, Microbubble, Attenuation, Contrast-to-tissue ratio, Pre-clinical, Decantation

## Abstract

The acoustic properties of two clinical (Definity, Lantheus Medical Imaging, North Billerica, MA, USA; SonoVue, Bracco S.P.A., Milan, Italy) and one pre-clinical (MicroMarker, untargeted, Bracco, Geneva, Switzerland; VisualSonics, Toronto, ON, Canada) ultrasound contrast agent were characterized using a broadband substitution technique over the ultrasound frequency range 12–43 MHz at 20 ± 1°C. At the same number concentration, the acoustic attenuation and contrast-to-tissue ratio of the three native ultrasound contrast agents are comparable at frequencies below 30 MHz, though their size distributions and encapsulated gases and shells differ. At frequencies above 30 MHz, native MicroMarker has higher attenuation values and contrast-to-tissue ratios than native Definity and SonoVue. Decantation was found to be an effective method to alter the size distribution and concentration of native clinical microbubble populations, enabling further contrast enhancement for specific pre-clinical applications.

## Introduction

High-frequency ultrasound (>20 MHz) is used to obtain high-spatial-resolution images for the imaging of intravascular structures (Rhee 2007), superficial tissues (Vogt et al. 2007) and pre-clinical animal models (Foster et al., 2011, Goertz et al., 2005, Sun et al., 2008) and in ophthalmology (Silverman et al. 2008).

Ultrasound contrast agents (UCAs) are gas-filled microbubbles (MBs) and are used clinically as blood pool tracers to significantly enhance the acoustic backscatter from blood. Definity (Lantheus Medical Imaging, North Billerica, MA, USA) and SonoVue (Bracco S.P.A., Milan, Italy) are two clinically licensed UCAs. MicroMarker (targeted and untargeted) (Bracco, Geneva, Switzerland; VisualSonics, Toronto, ON, Canada) is marketed as a pre-clinical UCA for contrast enhancement and molecular imaging in small animals. The fundamental backscatter response and duration of enhancement of Definity have been characterized in mice at 40 MHz at a peak negative pressure of 3.5 MPa (Sirsi et al. 2010); large Definity MBs (4–5 and 6–8 μm in diameter) were found to have longer persistence and stronger contrast enhancement than small MBs (1–2 μm in diameter). This study also concluded that dissolution of the gas core was the dominant mechanism of contrast decay and was larger than filtration and removal of UCAs by macrophages in the lung, liver and spleen. In another study, the concentration-dependent attenuation and backscatter properties of Definity at 30 MHz were also investigated, both *in vivo* and *in vitro;* the results suggested that doses between 10 and 60 μL kg^−1^ produce a linear increase in peak enhancement, and these doses were recommended for quantitative contrast flow studies in mice (Stapleton et al. 2009).

Attenuation of UCAs has previously been determined for *in vitro* experiments studying the fundamental acoustic response of UCAs: Definity over 12–29 MHz (Goertz et al. 2007) and over 5–15 MHz (Faez et al. 2011); SonoVue over 0.8–10 MHz (Gorce et al. 2000); untargeted MicroMarker over 18–25 MHz (Huo et al. 2010); and targeted MicroMarker over 4–13.5 MHz (Helfield and Goertz 2013). Data based on these results can be used to calculate the shell properties used in simulation studies of MB dynamics (Hoff 1996). Mean backscatter power and attenuation of lipid-encapsulated Sonazoid (Nycomed, Oslo, Norway), Definity and SonoVue and albumin-shelled Optison (Mallinckrodt, Hennef, Germany) were measured at 30 MHz as a function of concentration and time using an intravascular ultrasound transducer (Moran et al., 2002, Moran et al., 2005). The concentration range of 10^4^ to 10^6^ MBs mL^−1^ was found to present a linear relationship with mean backscatter power; however, these measurements were not corrected for attenuation caused by contrast in the intervening path.

According to the literature, alteration of the size distribution of MBs effectively changes the fundamental (Sirsi et al. 2010) and harmonic (Cheung et al., 2008, Goertz et al., 2003) responses when the MBs are insonated with high-frequency ultrasound. However, UCAs measured by various experimental setups give varying results for shell, gas and size distribution of MBs, and the differences in experimental methods cannot be excluded as having an effect on the measured acoustic response. Additionally, little work to date has discussed the acoustic response of sub-populations in which the boundary of the size distribution is determined based on consideration of the simulated resonant MB diameter at a specific applied frequency. Consequently, for pre-clinical applications at high frequencies, it would be of interest to compare the acoustics of three commercial UCAs using one experimental setup and determine how a specific size distribution influences the acoustic performance of MBs.

The aim of the work described here was to study the acoustic response at fundamental frequencies of three commercially available ultrasonic contrast agents—Definity, SonoVue and MicroMarker—as a function of size, using a pre-clinical ultrasound scanner over the frequency range 12–43 MHz at 20 ± 1°C. Based on the simulated resonant diameter, two sub-populations are formed by changing the size distribution through decantation of the original population of MBs. The analysis combining the measured size distribution and acoustic response of different populations addresses the question of which sub-population and which UCAs are most suitable for enhancing the fundamental response at ultrasound frequencies used for pre-clinical imaging.

## Methods

### Preparation of ultrasound contrast agents

Ultrasound contrast agents were reconstituted according to the manufacturer's guidelines and formed the “native MB population.” As the pre-reconstitution temperature of Definity influences acoustic properties (Helfield et al. 2012b), after removal from the fridge, Definity vials were left for 15 min to reach room temperature before activation. Based on the maximum concentration (Table 1) from the manufacturer's published literature, MB mixtures were diluted in air-saturated, distilled water at room temperature to reach the same concentration of 0.8 × 10^6^ MBs mL^−1^ and stirred for 1 min at 430 rpm before measurement. This concentration falls within the concentration regime previously reported to vary linearly at 30 MHz (Stapleton et al. 2009). The same dilution was employed in each sub-population of each UCA; the method used to obtain sub-populations is described below.

**Table 1:** Parameters and dilutions of the three ultrasound contrast agents

	Definity∗	SonoVue†	MicroMarker‡
Gas	C_3_F_8_	SF_6_	C_4_F_10_/N_2_
Shell	Phospholipid	Phospholipid	Polyethylene glycol, Phospholipids and fatty acid
Mean diameter (μm)§	1.1–3.3	2–3	2.3–2.9
Maximum concentration after reconstitution (microbubbles/mL)	1.2 × 10^10^	5 × 10^8^	2 × 10^9^
Dilution in this study	1:15,000	1:625	1:2500
Manufacturer	Lantheus Medical, North Billerica, MA, USA	Bracco, Milan, Italy	Bracco, Geneva, Switzerland; VisualSonics, Toronto, ON, Canada

∗ Lantheus Medical Imaging literature (2011).

† Gorce et al., 2000, Schneider, 1999.

‡ VisualSonics literature (2012).

§ Manufacturer's literature.

### Alteration of the size distribution of microbubbles by decantation

Microbubbles were divided into two sub-populations, referred to as large MBs and small MBs. This was achieved using the method of decantation, which has been found to be a simple method for altering the size distribution of MBs (Goertz et al., 2007, Gorce et al., 2000). For the specific decantation procedure, two parameters including MB size and decantation time were determined by calculating the resonance frequency and employing the Stokes equation.

**Fig. 1:**
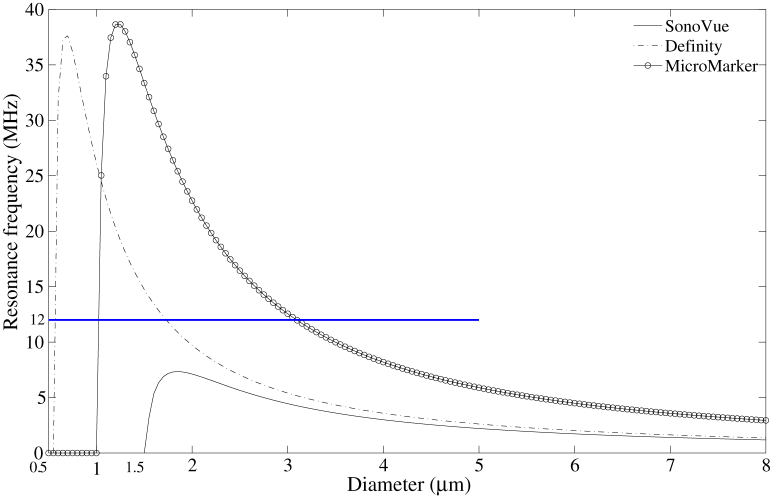
Simulated resonance frequency as a function of diameter for Definity, SonoVue and MicroMarker at 12-MHz driving frequency (derived from Goertz et al. [2007, eqn 7] using de Jong's model). The shell parameters of the three ultrasound contrast agents used in the simulation of resonance frequency are listed in Table 2.

The diameter used to separate the MBs into large and small populations was derived from the diameter of resonant MBs at 12 MHz, which is the lower limit of the frequency bandwidth used in this study. The simulated resonance frequencies of Definity, SonoVue and MicroMarker as a function of MB diameter are plotted in Figure 1 on the basis of results derived from Goertz et al. (2007) using de Jong's MB spherical oscillation model (de Jong et al. 1992). Shell parameters of the three UCAs used in the simulations are listed in Table 2. From these simulations, the critical size, defined as the size below which the encapsulated MBs would never resonate (Khismatullin 2004), was found to be 0.5 μm for Definity, 1.5 μm for SonoVue and 1 μm for MicroMarker when insonated at 12 MHz. Above the critical size, MBs with diameters less than 1.7 μm (Definity) and 3.1 μm (MicroMarker) resonate at frequencies higher than 12 MHz; no SonoVue MBs were found to resonate at frequencies higher than 12 MHz. To our knowledge, these simulations have not been confirmed by experimental observations. However, even if there is a small error associated with them, it is reasonable to assume the size of 2 μm to separate SonoVue and Definity MBs into small and large populations. In this study MicroMarker was not decanted.

**Table 2:** Shell stiffness, *S*_p_, and friction, *S*_f_, of the three UCAs used in the simulation

UCA	Measurement frequency range (MHz)	*S*_p_ (N m^-1^)	*S*_f_ (×10^−6^ kg s^−1^)	Reference
Definity	12–28	1.71 ± 0.24	0.015 ± 0.015	Goertz et al. (2007)
SonoVue	0.8–3	1.1	0.27	Gorce et al. (2000)
MicroMarker	18–25	10.3∗	0.15∗	Helfield et al., 2012a, Helfield et al., 2012b

UCA = ultrasound contrast agent.

∗ Shell elasticity χ = 5.15 N m^−1^ and shell viscosity κ_s_ = 3 × 10^−9^ kg s^−1^ (Helfield et al., 2012a, Helfield et al., 2012b) referred from Huo et al. (2010), *S*_p_ = 2χ, *S*_f_ = 16πκ_s_ (Doinikov and Bouakaz 2011).

The estimated decantation time for acquiring MBs of a specific diameter is derived from the Stokes equation, the principle of which is based on the varying times of different-sized MBs floating a certain distance (Goertz et al. 2007, eqn 12). The distance is determined by the required volume and the shape of the container used for decantation. In this study, 0.5 mL of 1-h-decanted Definity and 2 mL of 2-h-decanted SonoVue MBs were selected to ensure the same dilutions as their corresponding native populations. Collection of the decanted volume of UCA allows separation of the MBs that are <2 μm in diameter (“small MBs”); the remaining MBs are referred to as “large MBs” and are essentially the native solution after removal of small MBs. The details on decantation are given in Goertz et al. (2007). For the measurements described below, the same dilution was employed in each sub-population for the acoustic measurements.

### Measurement of size distribution and concentration

The size distributions of Definity, SonoVue and MicroMarker MBs were determined with a laser diffraction particle analyzer (Mastersizer 2000 Hydro MU, Malvern Instruments, Malvern, UK). The Mastersizer outputs the measured volume-based size distribution, and assuming the MBs are spherical, the concentration of MBs can be estimated. For measurements, the small and large MB populations were taken from the same vial. Native MBs were taken from a separate vial but from the same contrast agent batch. Three consecutive measurements were made for each contrast agent sample (in each sub-population: native, small and large).

### Experimental setup

**Fig. 2:**
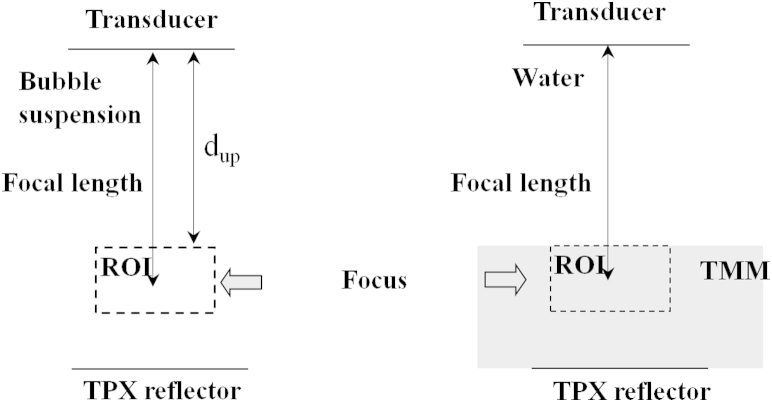
Experimental setup for calculation of contrast-to-tissue ratio measurement. (a) Backscattered signal from microbubble suspension. (b) Backscattered signal from tissue-mimicking material. *d*_up_ = distance between the transducer and the upper surface of the region of interest (ROI). TPX = polymethylpentene.

Experiments were performed based on a broadband substitution technique (AIUM 1995) using the radiofrequency (RF) data from a Vevo 770 pre-clinical ultrasound scanner (VisualSonics). This technique is described in more detail in Sun et al. (2012) and briefly summarized here. A circular water tank (7 cm in diameter, 4 cm in height) was placed on a mixing/magnetic stirrer (RCT Basic, IKA Works, Wilmington, NC, USA). A magnetic bar (3-mm o.d., 1 cm long) was placed in the tank to ensure a homogeneous MB suspension. A transducer was placed perpendicular to a polymethylpentene (TPX) reflector (Boedeker Plastics, Shiner, TX, USA). The focal position and region of interest (ROI) for the contrast-to-tissue ratio (CTR) measurement are illustrated in Figure 2.

Parameters of the four transducers (710B, 707B, 704 and 711) are listed in Table 3. Pulse length varied between 0.04 and 0.05 μs. As outlined in Table 3, the measured center frequency of the transducers was lower than that quoted by the manufacturer. The acoustic pressure from the four transducers was measured with a polyvinylidene fluoride membrane hydrophone (Precision Acoustics, Dorchester, UK) with a 0.2-mm-diameter active element. The acoustic pressure was measured over the range of output powers on the Vevo 770 scanner for each transducer. For the attenuation and CTR, the transmitting peak negative pressure (PNP) was set at 0.56 MPa in response to the minimal power level for transducers 710B and 707B. For transducers 704 and 711, the PNP was set at 0.58 MPa to approximate a PNP output comparable to that exhibited by transducers 710B and 707B.

**Table 3:** Characteristics of four high-frequency transducers∗

	RMV transducer model			
	710B	707B	704	711
Central frequency (MHz)	25	30	40	55
Focal length (mm)	15	12.7	6	6
Measured 3-dB bandwidth (MHz)	12–25	17–31	18–32	24–43
Measured central frequency (MHz)	18.5	24	25	33.5
Power	3%	3%	13%	50%
Peak negative pressure (MPa)	0.56	0.56	0.58	0.58

∗ The central frequency and focal length measurements are defined by the manufacturer's literature. The peak negative pressure was measured with a membrane hydrophone and the 3-dB bandwidth was measured from the frequency spectrum in response to the specific output power setting of each transducer.

### Attenuation measurement

The attenuation coefficient was calculated by subtraction of the frequency spectra of the RF signals from the TPX reflector with the MB suspension from the frequency spectra obtained with water in the tank (Coussios et al. 2004). The attenuation of the contrast agent suspensions was calculated over a reduced 3-dB frequency bandwidth of the transducers with a 5-MHz reduction at the lower frequency limit of each of the transducer bandwidths because of limited sensitivity. The attenuation coefficient α, in decibels per centimeter, was calculated using the equation

(1) $$\alpha =-\frac{10}{2d}{\text{log}}_{10}\left(\frac{I_{d}}{I_{0}}\right)$$

where *I*_0_ is the magnitude of the spectrum of the signal from the reflector with the water in the tank; *I*_*d*_ is the magnitude of the power spectrum of the signal from the reflector with the MBs in the tank; and *d* is the distance from the transducer to the upper surface of the TPX reflector and is equal to the focal depth. *D* is the distance taken from the RF data of the scanner assuming a speed of sound of 1540 m s^−1^. The speed of sound, *V*_water_, is from the published data (Bilaniuk and Wong 1993) at a specific temperature. The actual physical distance *d* between transducer and reflector is calculated as *d* = (*D*/1540) × *V*_water_.

Additionally, to determine whether the insonation PNP alters the acoustic properties of MBs, attenuation measurements using native Definity were repeated four times with 5-min intervals between scans in the same contrast sample using transducer 707B at the 3% power setting (PNP = 0.56 MPa).

For attenuation measurement, each transducer acquired 2700 measurements (three independent tests and 900 measurements per test), and the standard deviation was calculated from the mean results from the three independent tests.

### Calculation of contrast-to-tissue ratio

The CTR was calculated by normalizing the mean squared acoustic power of the backscattered signal from the MB suspension to the mean squared acoustic power of the backscattered signal of a tissue-mimicking material (Sun et al. 2012) placed at the focus of the transducer, as illustrated in Figure 2. The backscattered power was calculated from the power spectra of the received signal in frequency domain and integrated over the measured 3-dB bandwidth of the transducer (Table 3). Because the backscattered tissue-mimicking material signal was measured in water, the backscatter of the MB suspension was compensated by the attenuation of the ultrasound (α) through the MB suspension between the transducer and the upper surface of the ROI (*d*_up_).

(2) $$\text{CTR}=10\;{\text{log}}_{10}\left(\frac{\text{power}\;{\text{backscattered}}_{\text{bubble}}}{\text{power}\;{\text{backscattered}}_{\text{TMM}}}\right)+\alpha \cdot 2d_{\text{up}}$$

The RF data in the pre-selected ROI (1.05 × 1.6 mm) were saved and output, and MATLAB 2009A software (The MathWorks, Natick, MA, USA) was used for computational analysis. Each experiment was repeated three times and 900 independent samples (300 consecutive frames on three lines) in the ROI were collected per experiment. Between adjacent RF acquisition lines, the angle was less than 0.3° so the lines were assumed to be parallel. The RF data on the three lines were found to be independent from the autocorrelation calculations (data not included).

## Results

### Size distribution and concentration of microbubbles

**Fig. 3:**
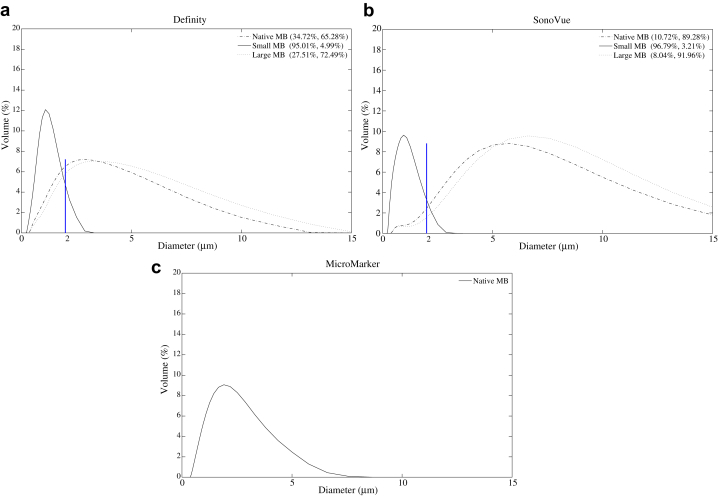
Volume-based size distributions of three populations of (a) Definity and (b) SonoVue and the single population of (c) MicroMarker. The two percentages in parentheses in (a) and (b) are the percentages below and above 2 μm. The maximum standard deviation of volume percentage was found to be less than 0.63%.

Figure 3 illustrates the volume-based size distributions of Definity, SonoVue and MicroMarker. The two percentages in the legends for Definity and SonoVue are the percentages of the native population that lie below and above 2 μm (the boundary). It can be seen that the small MBs are successfully removed *via* decantation to form the small MB population; however, small MBs still exist in the large MB population (volume percentage: 27.51% in Definity and 8.04% in SonoVue).

Table 4 outlines the variation in calculated concentrations of the two decanted subpopulations compared with the native population. For both Definity and SonoVue, the concentration of the small MB population was found to decrease, and the concentration of the large MB population was found to increase, after decantation.

**Table 4:** Variation in percentage of concentrations of small and large microbubbles compared with the native population∗

	Small microbubbles (%)	Large microbubbles (%)
Definity	−5.92 ± 0.74	8.12 ± 12.72
SonoVue	−23.05 ± 10.62	72.09 ± 11.74

∗ Negative and positive numbers indicate decreases and increases in concentration, respectively.

### Frequency-dependent attenuation of UCA suspensions

**Fig. 4:**
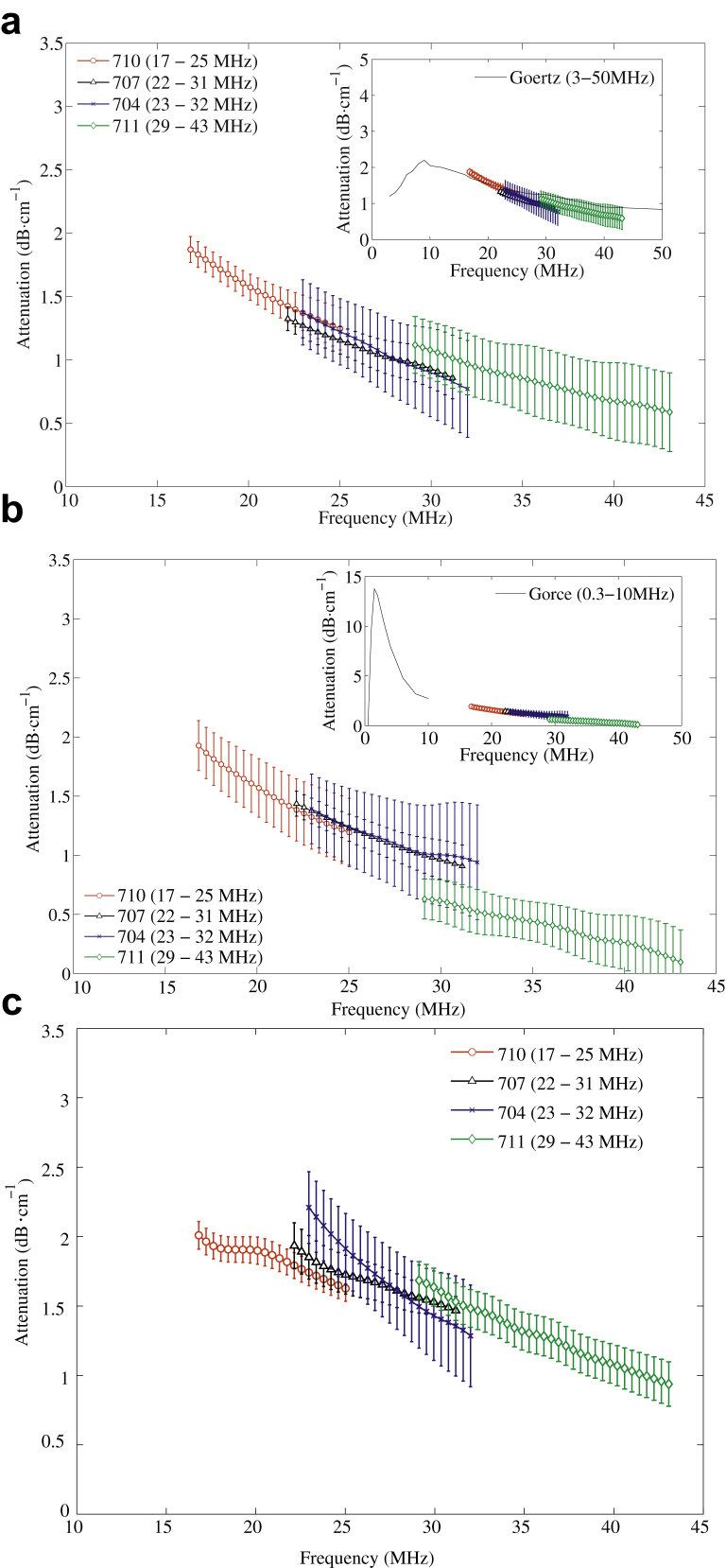
Frequency-dependent attenuation of native ultrasound contrast agents—(a) Definity, (b) SonoVue, (c) MicroMarker—measured by four transducers over their 3-dB bandwidths (5-MHz removal from the lower frequency end). (a, Inset) Attenuation of Definity is compared with the experimental results of Goertz et al. (2007). (b, Inset) Attenuation of SonoVue is compared with the results of Gorce et al. (2000). The concentration in Gorce and colleagues' study was 1:2000; therefore, the attenuation is corrected by multiplying by 3.2, as we used a 1:625 dilution. (c, Inset) MicroMarker. No attenuation curve has previously been published for MicroMarker. For the attenuation measurement, each transducer acquired 2700 measurements (three independent tests and 900 measurements per test), and the standard deviation was calculated from the means from the three independent tests.

Figure 4 illustrates attenuation (dB cm^−1^) as a function of frequency for the three UCAs. From the data collected in this study over the frequency range 17–43 MHz, attenuation decreases with increasing frequency for native Definity (Fig. 4a), SonoVue (Fig. 4b) and MicroMarker (Fig. 4c). The variation in attenuation over the frequency range 17–43 MHz for MicroMarker is the smallest and is less than 2 dB cm^−1^ in amplitude. The results are in good agreement with the published data for Definity (Goertz et al. 2007) and SonoVue (Gorce et al. 2000). Furthermore, the variation in the attenuation of ultrasound through native Definity suspension is found to be less than 0.5 dB cm^−1^ over the frequency range 17–31 MHz from the four intermittent measurements taken every 5 min.

### Contrast-to-tissue ratio

**Fig. 5:**
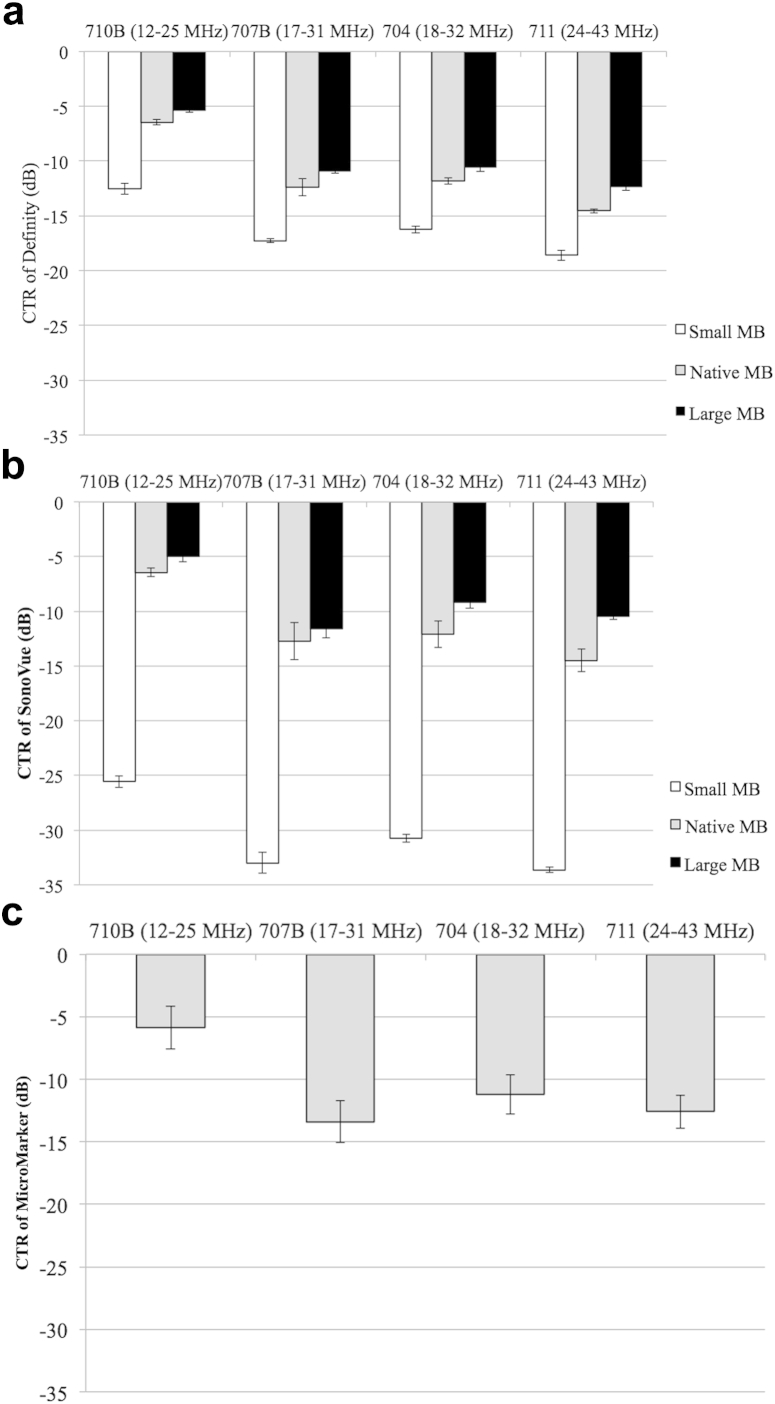
Contrast-to-tissue ratios of small, native and large populations of Definity and SonoVue and native population of MicroMarker measured by the four transducers over their 3-dB bandwidths. Error bars were calculated from three independent measurements.

Figure 5 illustrates the CTRs of the three populations (small, native and large) of SonoVue and Definity and of native MicroMarker measured by the four transducers. The CTR of each native UCA decreases more than 5 dB from transducer 710 B (12–25 MHz) to transducer 711 (24–43 MHz). The CTRs of Definity and SonoVue measured by transducers 707 B and 704 are comparable because of their similar driving frequency range. For each subpopulation of Definity and SonoVue, the large MB population produces the highest CTR. Despite the differences in compositions and size distributions, when the three native native UCAs are measured using the same transducer, the magnitudes of their CTRs are approximately the same.

## Discussion

### Influence of decantation

In this study, decantation was chosen to alter the size distribution of MBs. Its advantages over other methods such as filtration (Cheung et al., 2008, De Jong et al., 1992) and centrifugation (Feshitan et al., 2009, Choi et al., 2010, Streeter et al., 2010) are the ease of use of the technique, the lack of compression force applied and the ability to be able to preferentially select a size distribution of interest. Talu et al. (2008) and Barrack and Stride (2009) have reported the destruction of MBs using narrow-gauge needles (0.241 mm in diameter for 25-G, 0.159 mm in diameter for 30-G needle). MBs that either have been passed through a filter of diameter generally ranging between 1 and 10 μm or have experienced centrifugal forces are likely to be destroyed. Another drawback of using a filter is that the selection of MB size is directly restricted by the filter pore size from the manufacturer. Additionally, both filtration and centrifugation require pre-dilution of the agent. Specifically, MBs usually require high dilution (>1:1000), to allow sufficient space to isolate MBs in centrifugation and to avoid clogging in filtration pores. However, *in vivo* applications, especially small animal injections, typically require a limited injection volume (100 μL for mice [Foster et al. 2011]) and higher concentration (*e.g.,* MicroMarker 1 × 10^7^ MBs in 50 μL per injection) than laboratory *in vitro* experiments (*e.g.,* 0.8 × 10^6^ MBs/mL applied in this study). The only limitation of decantation is the time required to separate the different size distributions.

### Influence of pressure on attenuation measurement

Transmitting pressure for the acoustic measurement was set at 0.56 MPa (mechanical index [MI] = 0.16 at 12-MHz frequency) and 0.58 MPa (MI = 0.09 at 43-MHz frequency). Previous authors have found that small, individual Definity and MicroMarker MBs (<2 μm) are disrupted at 25 MHz using acoustic PNPs ranging from 0.2 to 1 MPa (Helfield et al. 2012a), that is, PNPs lower than that used in this study. However, from our experiments (data not included), we found that the attenuation of ultrasound through native Definity suspension does not vary with time over a 15-min period. This indicates that the applied PNP is non-destructive and does not change the acoustic properties of the MB suspension over the measurement period. Note that in published attenuation experiments, Goertz et al. (2007) used 25 kPa over the frequency range 12–29 MHz (maximal MI = 0.007) and Gorce et al. (2000) used 10 kPa over the frequency range 0.8–10 MHz (maximal MI = 0.011) as a transmitting pressure sufficient to produce a small oscillation of MBs. The attenuation measured at higher PNPs in this study is similar in magnitude to these published data. This implies that the attenuation of Definity and SonoVue displays a low sensitivity to pressure at these low non-destructive pressures over the frequency range 12–43 MHz. However, further investigations of pressure dependence are required to prove this hypothesis.

### Limitation of resonance frequency simulation

The attenuation values of native Definity (Fig. 4a) and SonoVue (Fig. 4b) measured over 17–43 MHz are consistent with published results at similar dilution ratios. De Jong et al. (1992) and Goertz et al. (2007) used values of the magnitude of attenuation to determine the shell stiffness and friction of MBs. The good agreement of the measured attenuation at high frequencies with the published results indicates the shell properties in Table 2 are appropriate for use in the simulations illustrated in Figure 1. However, the equations for the calculation of resonance frequency are derived from small-scale linear oscillations of encapsulated MBs, and the shell parameters in Table 2 are derived at frequencies different from the range of interest in this study; hence it is possible that the values of shell parameters may not be accurate. For example, 1-μm SonoVue was found to resonate at 21 MHz (Bouakaz and de Jong 2007), whereas according to Figure 1, 1-μm SonoVue was found to be below the critical size. However, the accuracy of the calculation of the resonant frequency is not an absolute requirement for this work. An approximate figure for the sizes of resonating and non-resonating MBs can be attained using the existing theory, and as indicated by the results, we consider that this is an acceptable means of guiding size fractionation.

### Influence of scattering cross section on contrast-to-tissue ratio

**Fig. 6:**
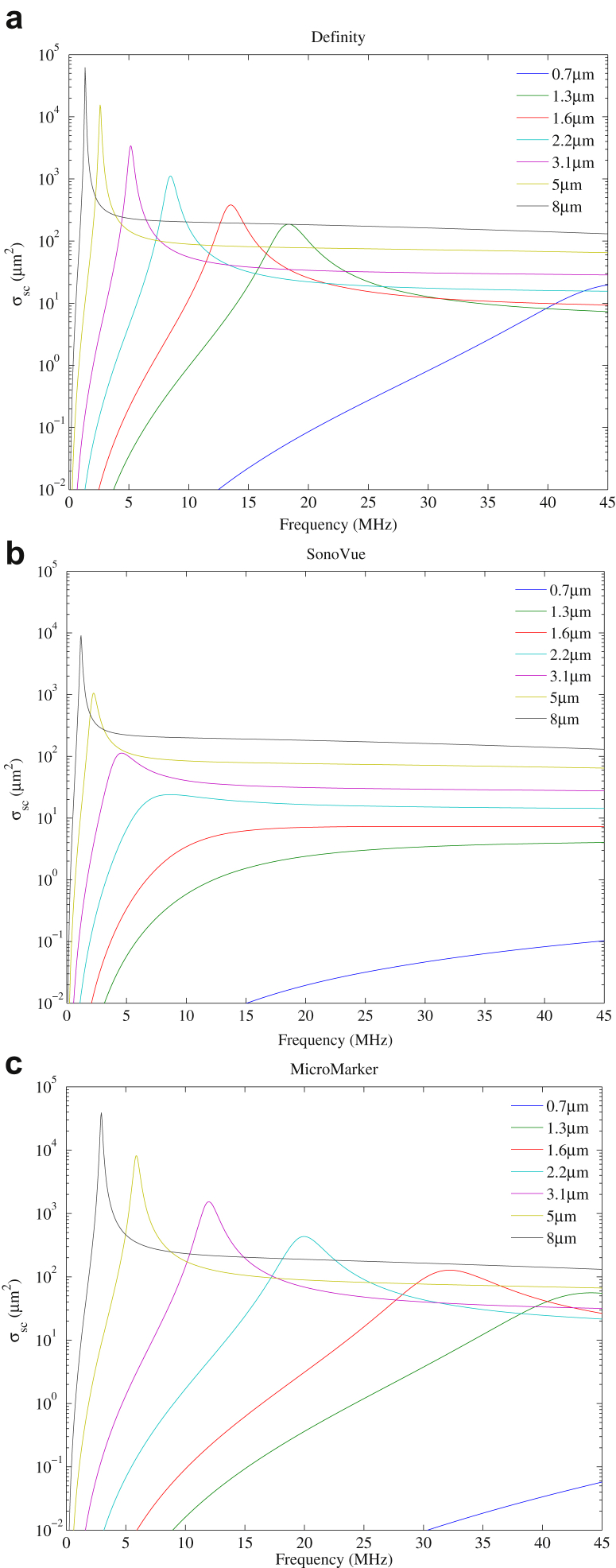
Simulation of scattering cross sections, σ_sc_, of (a) Definity, (b) SonoVue and (c) MicroMarker as a function of frequency at different diameters.

It was found from the simulated resonance frequency studies in Figure 1 that no SonoVue MBs resonated above 12 MHz. However, as illustrated in Figure 5, we found that the CTR of native SonoVue was comparable to that of Definity and MicroMarker over the high frequency range. A comparison of the scattering cross sections of the three UCAs may be helpful to consider. The simulations of scattering cross-section σ_sc_(*r*, *f*) in de Jong et al. (1992, eqn 1) are plotted in Figure 6, which gives the scattering cross sections of the three UCAs for different sizes in the vicinity of the estimated resonance diameters. It can be seen that over the frequency range 10–45 MHz, the scattering cross section of SonoVue microbubbles with diameter >2 μm is in the same range (10–10^3^ μm^2^) as Definity microbubbles of diameter >0.7 μm and MicroMarker microbubbles of diameter >0.7 μm. From Figure 3b, it is evident that the volume percentage of native SonoVue microbubbles is significantly greater than 2 μm, and hence the resultant scattering cross sections of the SonoVue native population may be comparable to those of Definity and MicroMarker.

### Impact of decantation on contrast-to-tissue ratio

For each UCA, the CTR of the native population lay between the responses from the small and large MB populations, with the largest CTR measured in the large MB population. Previous *in vivo* studies at 40 MHz found that different-size populations of Definity (4–5 μm in diameter, 6–8 μm in diameter) had a higher mean video intensity than Definity (1–2 μm in diameter), indicating that large MBs contributed predominately to the fundamental response (Sirsi et al. 2010). However, it should be noted that in this study, the Definity MBs in the small subpopulation are resonant MBs (<1.7 μm in diameter) above 12 MHz. The low CTR from the small resonant MBs suggests that at high frequency, the large off-resonance MBs are more suitable for enhancing the fundamental acoustic response than small resonant MBs, although the influence of decreasing concentration of small Definity MBs after decantation cannot be completely excluded. An improvement of 20 dB in the CTR of SonoVue large MBs over small MBs also supports this conclusion. Hence decantation can be an effective method for CTR enhancement at fundamental frequencies by increasing the absolute number of large MBs in a limited volume, which is particularly useful for practical *in vivo* pre-clinical injection regimes where the injected volume is often limited by animal tolerance.

In our study, we assume a concentration-matched test, and the data in Table 4 indicate the variation in percentage of concentration pre- and post-decantation. This is an estimation of the MB concentration from the Mastersizer. The experiments of volume-matched or cross section-matched boluses would also be of interest, but such studies require knowledge of the absolute number of microbubbles, which was beyond the measurement scope of the equipment used in this study.

### Comparison of three lipid UCAs and considerations of their pre-clinical applications

At the same concentration, the attenuation and CTR of the three native UCAs were found to be comparable when measured by transducers with frequency <30MHz (transducers 710B, 707B and 704) as illustrated in Figures 4 and 5. According to the results for transducer 711 (24–43 MHz), MicroMarker had a 2-dB increase in CTR and a larger attenuation of approximate 0.5–1 dB cm^−1^ at 30–43 MHz in comparison to native Definity and SonoVue. This indicates a likely advantage to the use of MicroMarker at frequencies >30 MHz. Future work will determine whether this agent is optimally imaged at harmonic frequencies. From the practical application point of view, at frequencies <30 MHz, not only Definity, but also SonoVue could be used in a pre-clinical setting at MB concentrations similar to those of MicroMarker. For instance, in mice, the recommended bolus injection of MicroMarker is 1 × 10^7^ MBs per 50 μL (1:10 dilution, 50 μL is the total volume of diluted bubble solution for injection) for cardiovascular, kidney and liver imaging and 1 × 10^8^ MBs per 50 μL for tumor, retina and hindlimb imaging, respectively. A 50-μL UCA injection of 1 × 10^7^ MBs is comparable to a 1:60 dilution for native Definity and 1:2.5 dilution for native SonoVue UCAs. However, for 1 × 10^8^ MBs per 50 μL, native SonoVue faces the dilemma of low concentration within limited injected volume (100 μL). As discussed, the advantages of using decanted large MBs for CTR enhancement may aid enhancement in pre-clinical injections. Thus, the practicalities of using SonoVue for high-frequency *in vivo* application require further studies, although the feasibility of using SonoVue has been found to be successful for imaging in mice at 6 MHz (Browning et al. 2011). Additionally, this study focused on the backscattered signals from MBs in the fundamental frequency range; the harmonic variation as a result of decantation would be of interest and requires further investigation.

## Conclusions

We characterized the acoustic attenuation and CTR of three lipid-encapsulated UCAs using high-frequency ultrasound from 12 to 43 MHz using a PNP of 0.56 MPa/0.58 MPa. At the same number concentration below 30 (results for transducers 710B, 707B and 704), the attenuation and CTR of native Definity, SonoVue and MicroMarker are comparable, though their size distributions and encapsulated gases and shells differ significantly. At frequencies >30 MHz (results for transducer 711 [24–43 MHz]), native MicroMarker produces a 0.5 dB cm^−1^ higher attenuation and a 2-dB higher CTR than native Definity and SonoVue. From the CTR comparison of sub-populations of Definity and SonoVue, altering the size distribution and concentration through decantation enables further enhancement for specific applications and may take full advantage of the imaging capabilities of high-frequency scanners for small animal applications.
